# Dengue Fever-Induced Complete Heart Block (Cardiac Dengue) Requiring Permanent Pacemaker Implantation in a 53-Year-Old Man: A Case Report

**DOI:** 10.7759/cureus.82372

**Published:** 2025-04-16

**Authors:** Maitha K Alnuaimi

**Affiliations:** 1 Internal Medicine, Tawam Hospital, Abu Dhabi, ARE

**Keywords:** bradyarrhythmia, cardiac complications, complete heart block, dengue fever, permanent pacemaker

## Abstract

Dengue fever is an endemic mosquito-borne viral infection in many tropical and subtropical regions. While its common manifestations include high-grade fever, rash, and myalgia, cardiac complications, particularly complete heart block, are rare but potentially life-threatening, underscoring the importance of vigilance. A 53-year-old man with type 2 diabetes mellitus and hypertension presented with dizziness, weakness, and a five-day history of high-grade fever with chills. He lived in a dengue-endemic area and denied any recent travel. Physical examination revealed bradyarrhythmia (38-49 beats per minute) and hypotension (99/72 millimeters of mercury). Laboratory tests confirmed acute dengue infection. Despite supportive care and temporary pacing, the patient’s intrinsic atrioventricular conduction showed no recovery, leading to persistent pacemaker dependence. On hospital day 17, a permanent dual-chamber pacemaker was implanted. He stabilized after the procedure and was discharged with close follow-up. The diagnosis of dengue was established using standard clinical criteria, confirmed by serologies for immunoglobulin M and immunoglobulin G and reverse transcription polymerase chain reaction. Comprehensive investigations excluded other bradyarrhythmia causes, such as Lyme disease, sarcoidosis, and myocarditis, supporting dengue as the sole etiology. This case highlights the rare but significant phenomenon of dengue-induced complete heart block, emphasizing that long-term pacemaker support may be necessary if conduction fails to recover. By adding to the limited data on dengue-associated advanced conduction disturbances, this case underscores the need for early recognition and multidisciplinary management to improve outcomes.

## Introduction

Dengue fever is a mosquito-borne illness caused by the dengue virus, which belongs to the Flaviviridae family. It is transmitted primarily by Aedes aegypti and Aedes albopictus mosquitoes and is endemic in over 100 countries, particularly in tropical and subtropical regions. According to the World Health Organization (WHO), approximately 100-400 million dengue infections occur annually, although the actual burden may be higher due to underreporting and misdiagnosis [1,2].

While the classic presentation includes high-grade fever, rash, headache, myalgia, and arthralgia, cardiac manifestations collectively termed ‘cardiac dengue' are increasingly recognized. This is likely due to enhanced diagnostic methods, a growing number of case reports, and emerging research on dengue’s cardiac involvement. The spectrum can range from benign arrhythmias to severe myocarditis, cardiogenic shock, and conduction disturbances [3]. Common arrhythmias in dengue include sinus bradycardia, atrial fibrillation, and junctional rhythms, most of which are transient [4]. Cardiac manifestations were observed in approximately 11-12% of dengue patients, with bradyarrhythmias being the most common (6.6%), predominantly presenting as asymptomatic sinus bradycardia. Notably, most arrhythmias resolved spontaneously within seven to 14 days [5]. However, complete heart block remains exceedingly rare, with only a few documented cases reported in diverse geographic regions and typically among patients with additional risk factors [6]. Proposed mechanisms for dengue-related conduction impairment encompass direct viral injury to the myocardium, immune-mediated inflammatory damage, and autonomic dysfunction [3,4]. Managing such cases often necessitates a multidisciplinary team, including specialists in cardiology, infectious diseases, and electrophysiology, to address both the cardiac and infectious aspects comprehensively.

In this report, we describe a 53-year-old man with persistent complete heart block secondary to dengue fever, ultimately requiring permanent pacemaker implantation. We discuss the potential pathophysiological mechanisms linking dengue infection to conduction system impairment, highlight the role of comorbidities in exacerbating disease severity, and emphasize the importance of a multidisciplinary approach to care.

## Case presentation

Diagnostic approach

Clinical assessment involved identifying key symptoms such as high-grade fever (often biphasic), headache, rash, and myalgias. Laboratory confirmation included testing for immunoglobulin M (IgM) and immunoglobulin G (IgG) antibodies against the dengue virus, in addition to reverse transcription polymerase chain reaction (RT-PCR). We utilized the WHO criteria for dengue diagnosis, which include clinical signs (fever, rash, myalgias) alongside positive laboratory findings. Nonstructural protein 1 (NS1) antigen testing was not performed, but the combination of IgM/IgG serologies and RT-PCR fulfilled diagnostic requirements. Additional investigations were conducted to exclude other possible etiologies, including negative blood and urine cultures, as well as negative serologies for human immunodeficiency virus (HIV), hepatitis A/B/C, and malaria.

Exclusion of other causes of bradyarrhythmia

A thorough medication review confirmed that the patient was not taking beta-blockers or other rate-limiting drugs. Electrolyte evaluations showed no significant disturbances aside from mild hyponatremia. Cardiac imaging with transthoracic echocardiography (TTE) and enzyme tests (cardiac troponin and N-terminal pro-B-type natriuretic peptide (NT-proBNP)) did not suggest an alternative etiology for complete heart block. We also considered other infectious causes of conduction disturbances (e.g., Chagas disease, Lyme carditis, and infective endocarditis) through region-specific epidemiological data and relevant serologies, which were negative. Furthermore, the patient’s clinical history, cardiac biomarker levels, and transthoracic echocardiographic findings did not support myocardial ischemia, infiltrative disease, or primary cardiomyopathy as alternative causes for his complete heart block. A cardiac MRI was not performed, as the patient’s critical condition and temporary pacemaker placement posed logistical and safety constraints. Computed tomography (CT) of the head excluded intracranial causes of bradyarrhythmia.

Initial presentation

A 53-year-old male with a medical history notable for type 2 diabetes mellitus and essential hypertension presented to the emergency department with complaints of dizziness, generalized weakness, and fatigue. He reported a five-day history of high-grade fever accompanied by chills and poor oral intake. He denied chest pain, shortness of breath, abdominal pain, headache, nausea, vomiting, allergen exposure, recent illnesses, or known mosquito exposure. On examination, he appeared slightly confused but was fully oriented in sensorium, with a Glasgow Coma Scale score of 15/15. This mild confusion was most likely multifactorial, related to fever and relative hypotension; a formal neurological consultation was not pursued because the patient’s mentation improved once hemodynamic stability was achieved.

Vital signs on admission revealed bradyarrhythmia (heart rate ranging from 38 to 49 beats per minute), blood pressure of 99/72 mmHg, respiratory rate of 20-23 breaths per minute, temperature of 37.7°C, and oxygen saturation of 95% on room air. Physical examination was otherwise unremarkable, and systemic examination did not reveal any focal abnormalities. There was no evidence of peripheral edema or signs suggestive of heart failure. The patient was not on any rate-limiting medications. Below is a summary of key laboratory results upon admission, highlighting notable abnormalities such as thrombocytopenia, elevated liver enzymes, and elevated cardiac biomarkers (Table 1).

**Table 1:** Admission laboratory investigations

Parameter	Result	Reference Range
Platelet count (×10^9/L)	61	150–450
Sodium (millimoles per liter)	133	135–145
Aspartate aminotransferase (AST, units/L)	368	10–40
Alanine aminotransferase (ALT, units/L)	306	10–40
N-terminal pro-B-type natriuretic peptide	1,630	<125 (most <75 y/o)
High-sensitivity troponin T (nanograms/L)	58–62.7	<14
C-reactive protein (CRP, milligrams/L)	13.9	<5
Hemoglobin (grams/L)	180	130–170 (male)
Red blood cell Count (×10^12/L)	6.39	4.5–5.9 (male)
pH	7.44	7.35–7.45
Partial pressure of carbon dioxide (pCO₂, mmHg)	29	35–45
Partial pressure of oxygen (pO₂, mmHg)	64	80–100
Bicarbonate (HCO₃⁻, millimoles per liter)	20	22–26

Dengue virus serology was positive for both IgM and IgG antibodies; RT-PCR confirmed the diagnosis of acute dengue infection. Tests for HIV, hepatitis A/B/C, and malaria were negative. Blood and urine cultures showed no bacterial growth.

**Figure 1:**
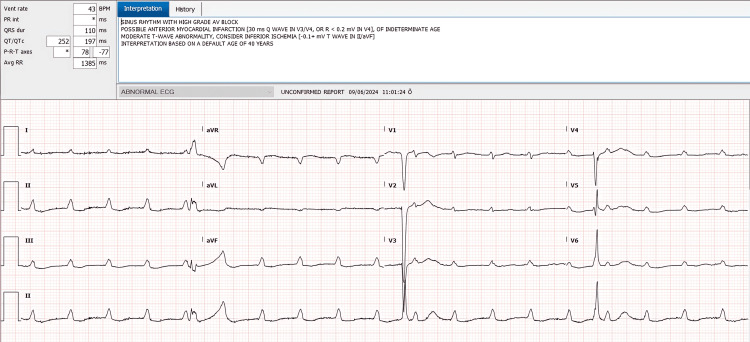
Admission ECG showing complete atrioventricular block with a ventricular rate of 38 bpm and T-wave abnormalities bpm: beats per minute

Upon admission, an electrocardiogram (ECG) was obtained to evaluate the nature of the bradyarrhythmia, revealing a complete atrioventricular block and significant T-wave changes (Figure 1).

Hospital course and pacing management

Given the hemodynamic instability due to significant bradyarrhythmia, the patient was admitted to the intensive care unit (ICU). The infectious disease consult team and general internal medicine were involved, along with cardiology, to guide comprehensive management. Despite pharmacological intervention, the patient’s heart rate remained between 38 and 49 bpm. 

**Figure 2:**
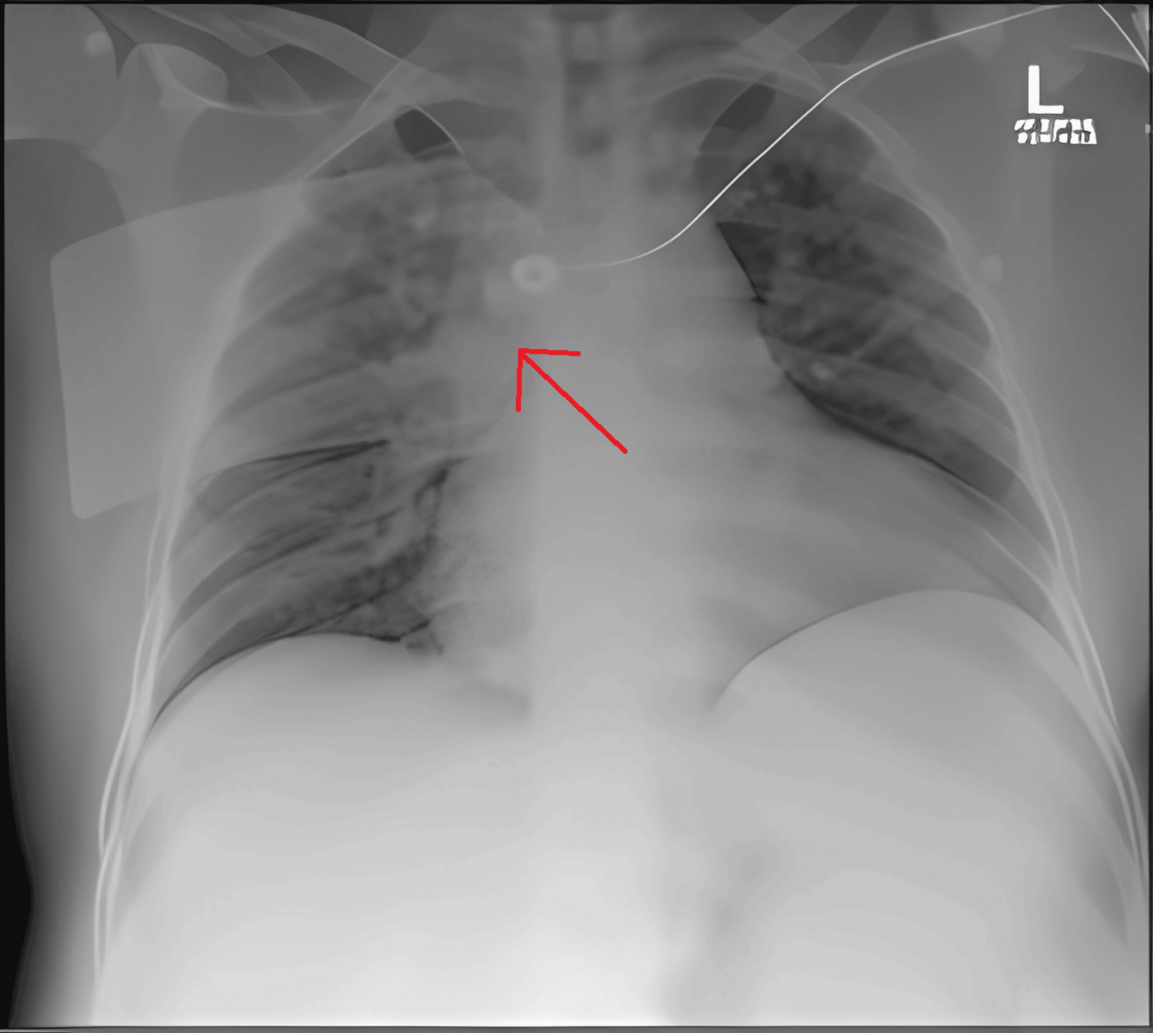
Chest X‐ray demonstrating the temporary pacing lead within the right ventricle Chest X-ray demonstrating the faint temporary pacing lead (arrow) within the right ventricle. The lead can be seen tracing from the left subclavian area across the midline into the right ventricle.

A temporary transvenous pacemaker was placed due to persistent bradyarrhythmia and hypotension. A chest radiograph was obtained to evaluate the position of the temporary pacing lead, confirming its location in the right ventricle (Figure 2). TTE demonstrated a normal-sized left ventricle with mild systolic dysfunction (left ventricular ejection fraction (LVEF) of 40%-45%), mild tricuspid regurgitation, and no significant regional wall motion abnormalities. Although elevated troponin and NT-proBNP suggested myocardial involvement, advanced imaging (e.g., cardiac MRI) was not pursued, given the patient’s critical condition and the presence of a temporary pacemaker. Monitoring of the pacing thresholds showed no recovery of intrinsic conduction when temporary pacing output was intermittently lowered to assess for junctional or sinus activity.

**Figure 3:**
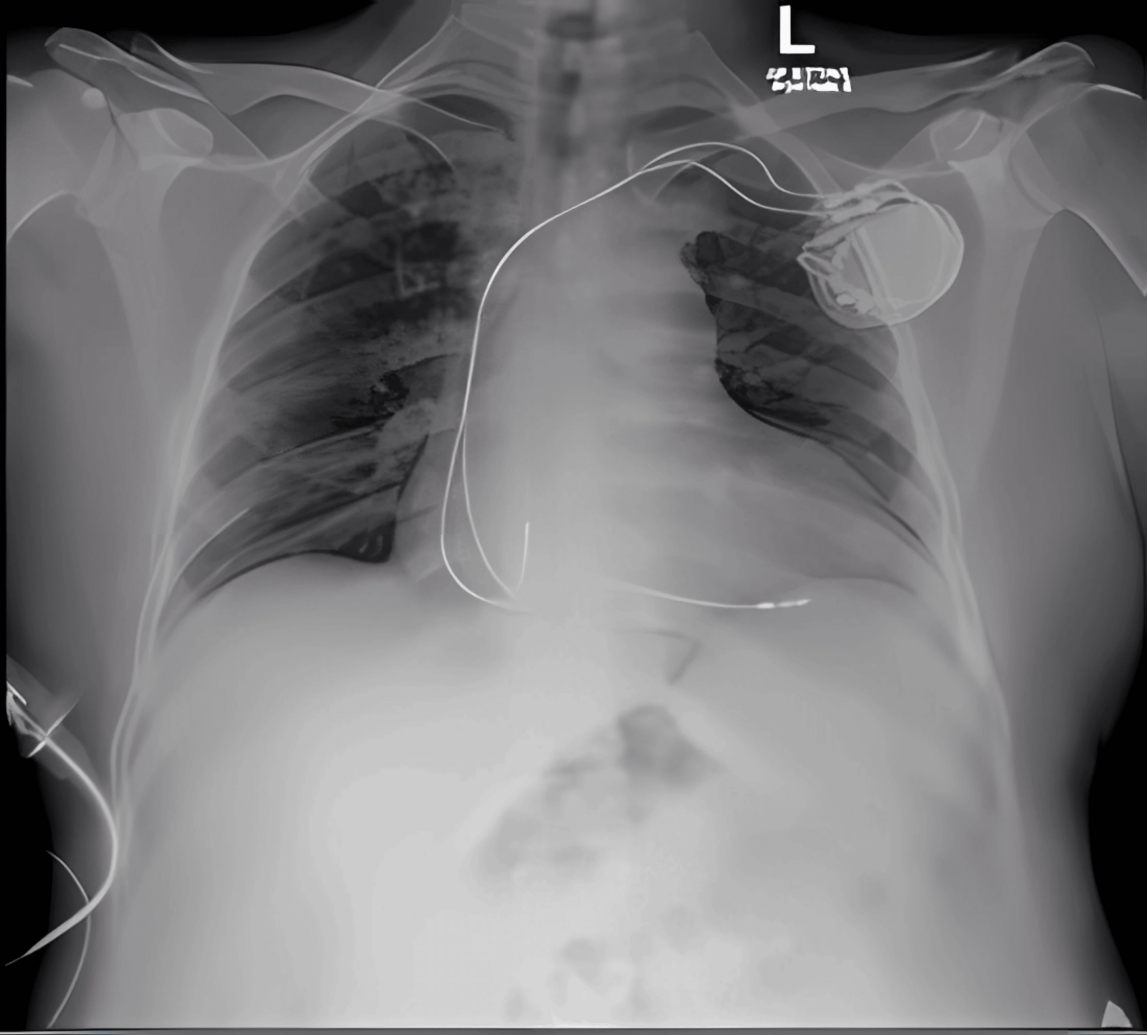
Serial chest radiograph demonstrating stable pacing lead position and no device‐related complications

Despite intravenous dopamine, the heart rate remained in the 38-49 bpm range, and serial electrocardiograms consistently demonstrated persistent complete heart block without recovery of intrinsic atrioventricular (AV) conduction. Serial chest radiographs confirmed a stable lead position with no device-related complications (Figure 3). His underlying junctional escape rhythm was inadequate (averaging 33-35 bpm) and did not improve over time.

**Figure 4:**
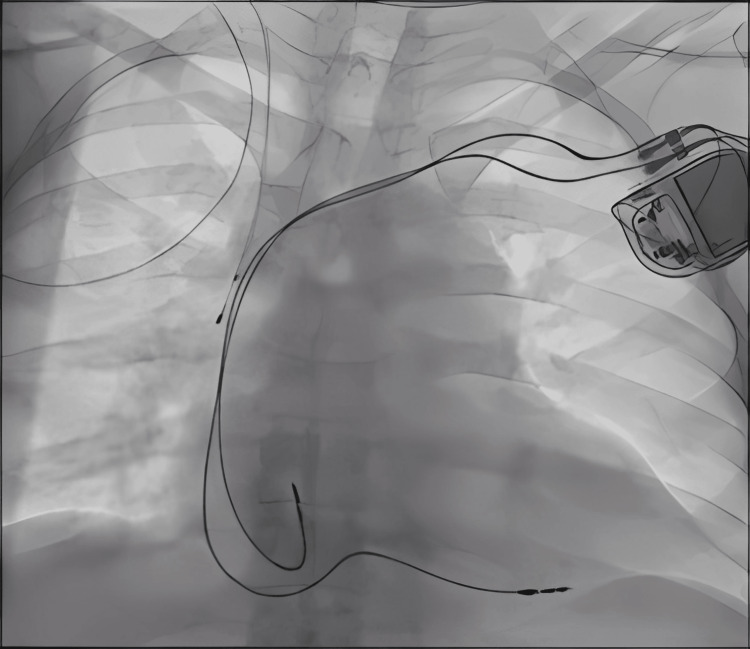
Post-permanent pacemaker insertion chest radiograph confirming proper lead placement

**Figure 5:**
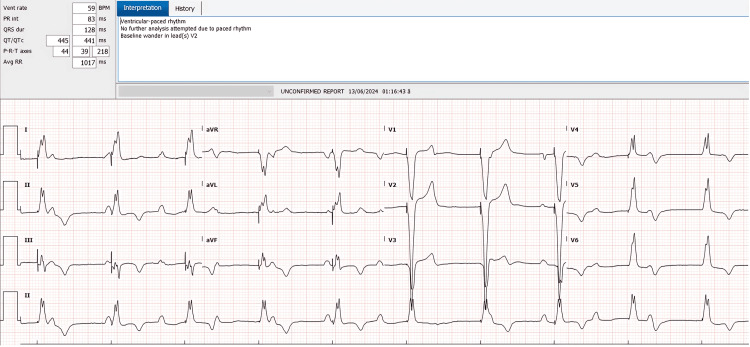
Post‐procedure ECG confirming stable dual‐chamber pacing and no intrinsic AV conduction AV: atrioventricular

On hospital day 17, a decision was made to proceed with permanent pacemaker implantation. Under local anesthesia, a dual-chamber permanent pacemaker (Enitra DR, BIOTRONIK SE and Co. KG, Berlin, Germany) was inserted. Following the procedure, the patient’s condition stabilized, and he was subsequently weaned off inotropic support. A post-procedure chest radiograph confirmed proper lead placement of the permanent pacemaker (Figure 4), and a follow-up ECG showed stable dual-chamber pacing with no intrinsic AV conduction (Figure 5). Subsequently, the patient’s fever resolved, liver enzymes and platelet counts improved, and he was discharged on hospital day 18 with instructions on pacemaker care and activity restrictions.

**Figure 6:**
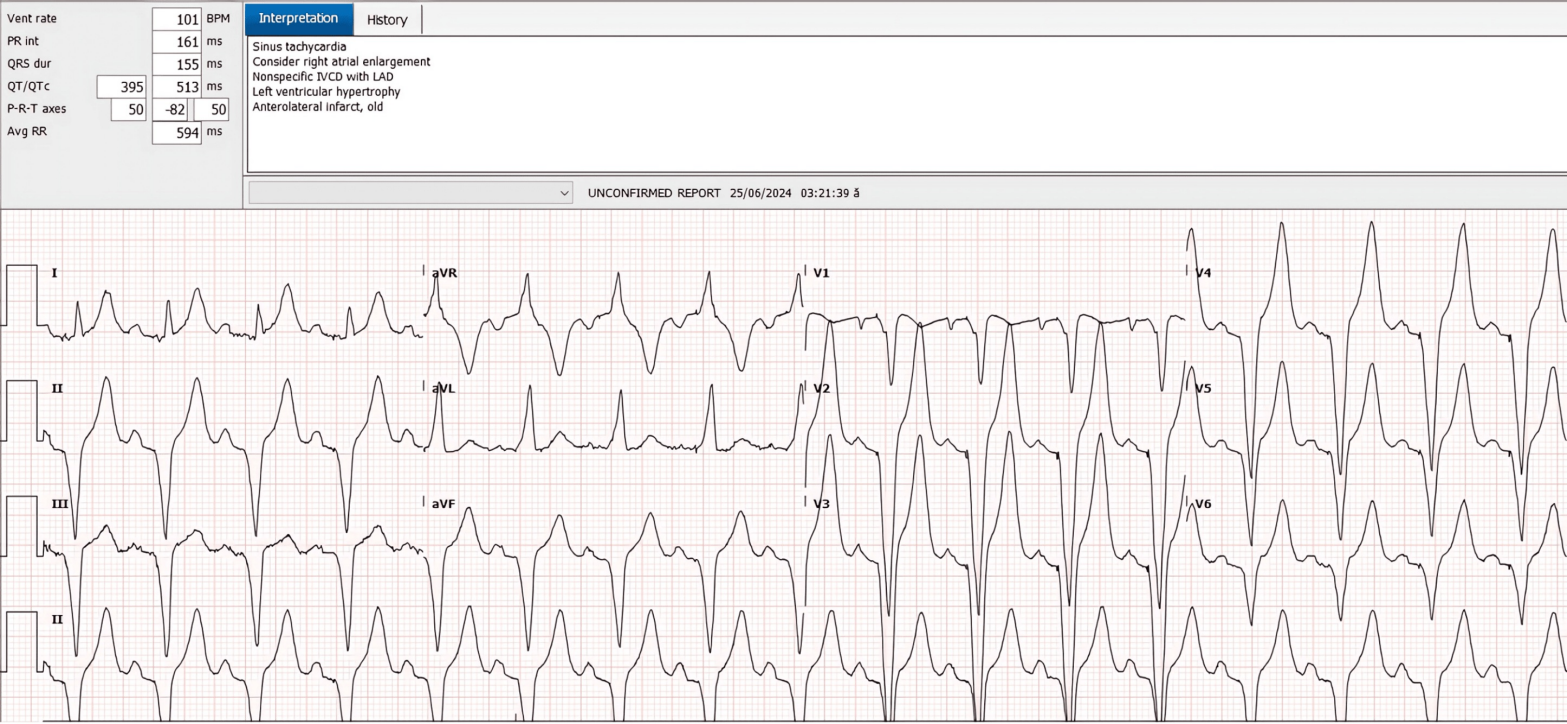
Discharge ECG demonstrating stable dual-chamber paced rhythm without any spontaneous conduction

An ECG at the time of discharge verified appropriate pacemaker function and the absence of intrinsic conduction (Figure 6). Follow-up in the cardiology clinic was arranged for two weeks post-discharge.

## Discussion

Cardiac involvement in dengue, often referred to as “cardiac dengue,” can present as a range of arrhythmias, both bradyarrhythmias and tachyarrhythmias, as well as myocarditis or pericarditis [7]. Although mild AV conduction disturbances are relatively common, persistent complete heart block requiring permanent pacemaker implantation remains exceedingly rare [6,8]. Several mechanisms have been proposed to explain such cardiac complications, including direct viral injury to cardiomyocytes, immune-mediated damage triggered by cytokine release, microvascular dysfunction owing to increased vascular permeability, and autoimmune responses that may mimic or directly target cardiac tissue [4].

Direct viral injury to the conduction system may result from the virus invading specialized nodal or His-Purkinje fibers, causing focal inflammation or necrosis that disrupts normal impulse propagation. Immune-mediated damage triggered by cytokine storms can extend to these conduction tissues, creating edema or scarring that impairs electrical conduction [7]. Microvascular dysfunction, often characterized by vascular leakage in dengue, can further compromise perfusion of the conduction system, exacerbating ischemic damage. Autoimmune responses, potentially arising from molecular mimicry, may also lead to antibody- or cell-mediated attacks on conduction tissue. While large-scale biopsy data are limited, certain autopsy and histopathological reports in severe dengue support these mechanisms by showing inflammatory infiltrates and hemorrhagic lesions in both the working myocardium and specialized conduction pathways [9].

The presence of comorbid conditions, such as diabetes mellitus and hypertension, could have predisposed our patient to endothelial dysfunction and possibly amplified the severity of his dengue-related cardiac injury [10]. Diabetes in particular impairs microvascular repair and promotes a pro-inflammatory state, potentially worsening endothelial damage within the conduction system. Hypertension may further exacerbate microvascular stress, collectively increasing the risk of irreversible conduction block. Chronic illnesses can weaken host immune defenses and worsen the inflammatory response, potentially contributing to persistent conduction disturbances, as observed in this case. Recognizing the higher risk in patients with comorbidities is critical for the timely identification of cardiac complications in dengue.

A multidisciplinary approach is essential to manage these complex presentations effectively. Involvement of infectious disease specialists, cardiologists, intensivists, and general internists allows for comprehensive care that addresses both the infectious and cardiologic aspects of severe dengue. Nevertheless, certain limitations can arise, such as the inability to perform advanced imaging like cardiac MRI when a temporary pacemaker is in situ. Additionally, there is currently no widely available specific antiviral therapy for dengue, emphasizing the need for supportive treatment while research into investigational antivirals or immunomodulatory agents continues. Therapies such as corticosteroids or IVIG have been explored anecdotally in severe dengue myocarditis, but robust data are lacking, and their specific benefit in conduction disturbances is unproven. Beta-agonists (e.g., isoproterenol) may transiently support heart rate but do not address the underlying viral or inflammatory injury. Colchicine, often used in inflammatory cardiac conditions, has not been systematically studied in dengue-induced AV block. While immunomodulators (such as IL-6 inhibitors or tumor necrosis factor alpha (TNF-alpha) blockers) show promise in other inflammatory conditions, their use in severe dengue remains investigational, with limited preliminary data and concern about potential hemorrhagic complications.

Early recognition of cardiac involvement is vital to improving outcomes. Biomarkers such as troponin, C-reactive protein (CRP), and NT-proBNP can help detect myocardial injury and systemic inflammation, guiding clinicians toward prompt interventions. Moreover, testing for NS1 antigen early in the disease course may facilitate timely diagnosis and management, potentially preventing severe complications [11]. To better characterize dengue-associated cardiac complications, especially conduction block, multinational registries or collaborative databases would be invaluable. Although there is no widely recognized global registry focused specifically on dengue myocarditis or conduction disorders, expanding existing dengue surveillance to capture cardiac endpoints could help clarify true incidence, risk factors, and outcomes.

Future directions in research include the development of targeted antiviral therapies that could reduce the viral load and associated inflammatory damage, thereby mitigating the risk of complete heart block. Larger cohort studies are also needed to determine the true incidence of permanent conduction disturbances in dengue and to clarify risk factors that predispose patients to irreversible conduction system damage. This case underscores a rare but severe cardiac manifestation of dengue fever. Persistent complete heart block necessitating permanent pacemaker implantation is an unusual outcome and highlights the need for clinicians to remain vigilant for bradyarrhythmias in dengue. Early diagnosis, close monitoring of cardiac function, and a multidisciplinary approach are crucial for improving patient outcomes. Enhanced research efforts, particularly regarding specific antiviral or immunomodulatory therapies, may further reduce morbidity and mortality associated with “cardiac dengue.”

## Conclusions

This case highlights a rare yet severe cardiac manifestation of dengue fever, with persistent complete heart block requiring permanent pacemaker implantation. It underscores the need for earlier and more frequent cardiac monitoring, using routine ECGs and serial biomarkers (e.g., troponin, NT-proBNP), especially in high-risk patients. Our findings suggest that conduction disturbances in dengue may not always be transient, raising the possibility that persistent AV block could be more common than currently recognized. Further research is warranted to clarify the timeline of AV block resolution and to evaluate targeted antiviral or immunomodulatory therapies, guiding future management strategies.
